# Two isoforms of TALDO1 generated by alternative translational initiation show differential nucleocytoplasmic distribution to regulate the global metabolic network

**DOI:** 10.1038/srep34648

**Published:** 2016-10-05

**Authors:** Tetsuji Moriyama, Shu Tanaka, Yasumune Nakayama, Masahiro Fukumoto, Kenji Tsujimura, Kohji Yamada, Takeshi Bamba, Yoshihiro Yoneda, Eiichiro Fukusaki, Masahiro Oka

**Affiliations:** 1 Laboratory of Nuclear Transport Dynamics, National Institutes of Biomedical Innovation, Health and Nutrition (NIBIOHN), 7-6-8 Saito-Asagi, Ibaraki, Osaka 567-0085, Japan; 2 Department of Frontier Biosciences, Graduate School of Frontier Biosciences, Osaka University, 1-3 Yamada-oka, Suita, Osaka 565-0871, Japan; 3 Department of Biotechnology, Graduate School of Engineering, Osaka University, 2-1 Yamada-oka, Suita, 565-0871, Japan; 4 NIBIOHN, 7-6-8 Saito-Asagi, Ibaraki, Osaka 567-0085, Japan; 5 Laboratory of Nuclear Transport Dynamics, Graduate School of Pharmaceutical Sciences, Osaka University, 1-6 Yamada-oka, Suita, Osaka 565-0871, Japan

## Abstract

Transaldolase 1 (TALDO1) is a rate-limiting enzyme involved in the pentose phosphate pathway, which is traditionally thought to occur in the cytoplasm. In this study, we found that the gene *TALDO1* has two translational initiation sites, generating two isoforms that differ by the presence of the first 10 N-terminal amino acids. Notably, the long and short isoforms were differentially localised to the cell nucleus and cytoplasm, respectively. Pull-down and *in vitro* transport assays showed that the long isoform, unlike the short one, binds to importin α and is actively transported into the nucleus in an importin α/β-dependent manner, demonstrating that the 10 N-terminal amino acids are essential for its nuclear localisation. Additionally, we found that these two isoforms can form homo- and/or hetero-dimers with different localisation dynamics. A metabolite analysis revealed that the subcellular localisation of TALDO1 is not crucial for its activity in the pentose phosphate pathway. However, the expression of these two isoforms differentially affected the levels of various metabolites, including components of the tricarboxylic acid cycle, nucleotides, and sugars. These results demonstrate that the nucleocytoplasmic distribution of TALDO1, modulated via alternative translational initiation and dimer formation, plays an important role in a wide range of metabolic networks.

Metabolism is a crucial process for the survival of organisms that generates both energy and biological materials. In general, metabolic pathways often proceed in their specific subcellular compartments. For example, glycogenesis is known to take place in the cytoplasm, while the tricarboxylic acid (TCA) cycle occurs in the mitochondria^1^. Therefore, metabolic enzymes, which catalyse various chemical reactions involving their target metabolites, localise in appropriate subcellular compartments to achieve their specific functions.

The pentose phosphate pathway, which branches off from glycolysis, generates nicotinamide adenine dinucleotide phosphate (NADPH), which serves as a reducing reagent in redox reactions, and ribose-5-phosphate (R5P), which is used in nucleotide synthesis. The pathway consists of two distinct phases, an oxidative phase that allows reduction of NADP^+^ to NADPH, and a non-oxidative phase in which R5P is reversibly converted to glycolytic intermediates such as glyceraldehyde-3-phosphate (GAP) and fructose-6-phosphate (F6P). The pentose phosphate pathway is believed to proceed exclusively in the cytoplasm^2^. However, our previous proteomic analysis identified transaldolase 1 (TALDO1), one of the pentose phosphate pathway enzymes, as a nuclear protein that interacts with importin α^3^, a nuclear import receptor that recognises a nuclear localisation signal (NLS). Furthermore, we showed that the nucleocytoplasmic transport of TALDO1 is actively regulated via the classical importin α/β-dependent import pathway and the CRM1-dependent export pathway^3^. However, the regulatory mechanism and functional significance of its nucleocytoplasmic shuttling remain unknown.

TALDO1 catalyses the conversion of sedheptulose-7-phosphate (S7P) and GAP into erythrose-4-phosphate and F6P^4^, and functions as a rate-limiting enzyme of a non-oxidative phase in the pentose phosphate pathway^5^. Indeed, a lack of TALDO1 causes the upregulation of S7P^6^ and downregulation of NADPH and glutathione^7^,^8^,^9^. Furthermore, the malfunction of TALDO1 is known to cause disease; TALDO1-deficient patients present with a distinct set of symptoms in the liver such as signs of fibrosis, cirrhosis, and cholestasis, resulting from the damage to hepatocytes and intrahepatic biliary cells^2^,^10^,^11^. Additionally, it has been reported that TALDO1 expression is increased in tumours^12^,^13^. In cancer cells, TALDO1 plays critical roles in accelerating cell proliferation by supplying R5P for nucleic acid synthesis and NADPH for both the synthesis of fatty acids and cell survival, especially under stress conditions^7^,^14^,^15^. Taken together, these observations suggest that the nuclear:cytoplasmic concentration ratio of TALDO1, which is most likely modulated via its nucleocytoplasmic shuttling, is critical for the regulation of the pentose phosphate pathway.

In this study, we have demonstrated that two isoforms of TALDO1 are generated via alternative translational initiation, and they exhibit differential nucleocytoplasmic localisation. Furthermore, our metabolic analysis revealed that, unexpectedly, the nucleocytoplasmic distribution of TALDO1 affects a broad range of metabolic pathways other than the pentose phosphate pathway alone.

## Results

### *TALDO1* has two translation initiation sites

**Figure 1:**
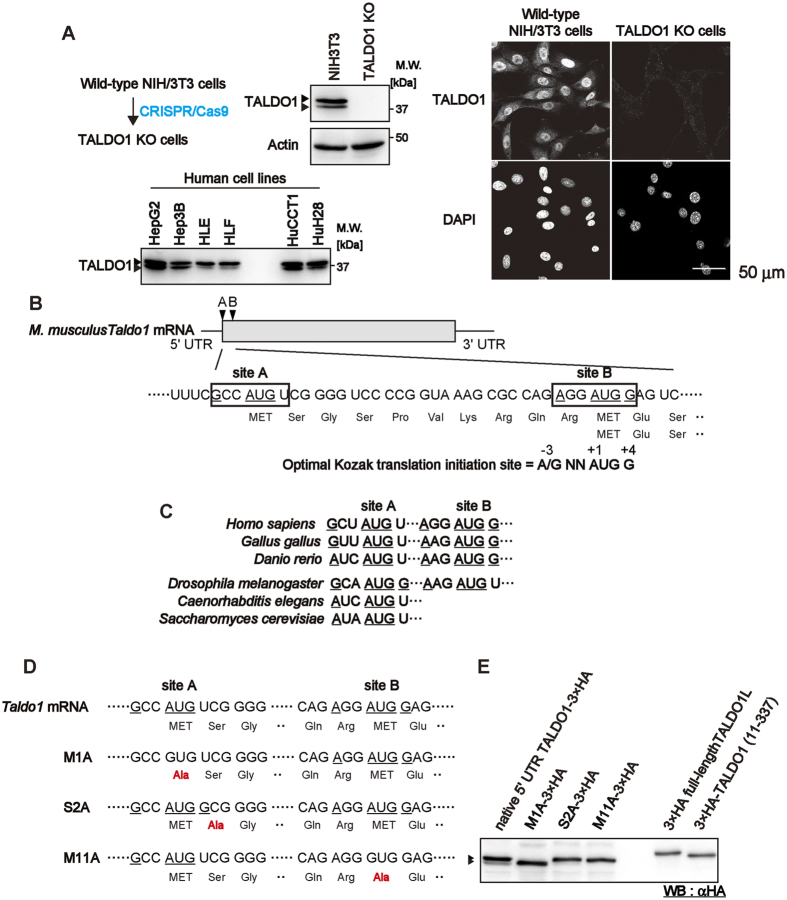
*TALDO1* has two translation initiation sites. (**A**) TALDO1 isoforms were analysed by western blotting in NIH/3T3 (wild-type and TALDO1 knockout [KO]) and human cell lines (HepG2, Hep3B, HLE, HLF, HuCCT1, and HuH28 cells). Three and a half micrograms (NIH/3T3) or 27.6 μg (human cell lines) of total protein was loaded in each lane for sodium dodecyl sulphate-polyacrylamide gel electrophoresis. Actin was used as a loading control. Immunofluorescence images of wild-type NIH/3T3 and TALDO1 KO cells stained with anti-TALDO1 antibody. Cell nuclei were stained using DAPI. (**B**) Schematic representation of the conserved *Mus musculus* (mouse) *Taldo1* sequence around two translation initiation sites. Two potential in-frame translation initiation sites, designated sites A and B, are present in the *Taldo1* mRNA. Underlined nucleotides indicated potential translation initiation AUG-sites and critical nucleotides at positions −3 and +4 according to the optimal Kozak translation initiation consensus sequence. (**C**) Comparison of the region of *Taldo1* sequence around two translation initiation sites from *Homo sapiens* (human), *Gallus gallus* (chicken), *Danio rerio* (zebrafish), *Drosophila melanogaster* (fruitfly), *Caenorhabditis elegans* (nematode), and *Saccharomyces cerevisiae* (yeast). (**D**) Schematic representations of the native and mutant 5′ untranslated regions (UTRs) of the *Taldo1* gene used in this study. (**E**) Western blot using anti-HA antibody showing the expression of native 5′ UTR-TALDO1-3×HA, M1A-3×HA, S2A-3×HA, M11A-3×HA, 3×HA-TALDO1L, and 3×HA-TALDO1S transfected into wild-type NIH/3T3 cells.

When we performed western blotting using an anti-TALDO1 antibody, we noticed that the antibody detected two bands around 37 kDa (the estimated molecular weight of TALDO1 is 37.39 kDa) in cell lysates prepared from mouse and human cell lines (Fig. 1A). The expression levels and the ratio of the two bands varied among the cell lines. To confirm whether these two bands were TALDO1 proteins, we produced TALDO1 knockout (KO) mouse NIH/3T3 cells using CRISPR/Cas9-mediated genome engineering (Supplementary Figure 1). As shown in Fig. 1A, lane 2, neither of these bands were detected in the lysates of TALDO1 KO cells, confirming that they were TALDO1 proteins. Additionally, immunofluorescence analysis revealed the loss of nuclear staining of TALDO1 in the TALDO1 KO cells.

Notably, two potential in-frame translation initiation sites denoted as sites A and B exist in mouse *Taldo1* mRNA (Fig. 1B), and these sites are highly conserved in higher eukaryotes (Fig. 1C). It is well known that the sequence flanking the AUG initiation codon (Kozak sequence) plays an important role in the enhancement of translational initiation by eukaryotic ribosomes^16^. Furthermore, the initiation of alternative translation is, in some cases, dependent on the strength of the codon-anticodon interaction^17^. Indeed, we found that the Kozak sequence within site A of *TALDO1* contains a U instead of a G at position +4, suggesting that it is not optimal for efficient initiation. In contrast, site B contains a perfect Kozak consensus sequence. Furthermore, TALDO1 was listed as a protein that potentially contains an alternative translational initiation site in previous reports^18^,^19^. Taken together, these observations suggest that an alternative translation of TALDO1 starts from the second methionine at amino acid (A.A.) position 11, causing the generation of a short isoform of TALDO1 lacking the first 10 N-terminal amino acids.

To corroborate this hypothesis, a C-terminal triple haemagglutinin (3×HA)-tagged TALDO1 containing an intact 5′ untranslated region (UTR) (native 5′ UTR TALDO1-3×HA) was expressed in wild-type NIH/3T3 cells, and immunoblotting was performed using an anti-HA antibody. As shown in Fig. 1E (first lane), two bands, which likely resulted from the alternative translational initiation, were observed. Next, when the N-terminal 3×HA-tagged full-length TALDO1 was expressed; it was detected as a single protein (Fig. 1E, right panel), demonstrating that TALDO1 is not truncated by post-translational modification or proteolysis. Furthermore, the mutant lacking the first 10 amino acids of TALDO1 (11–337) was also detected as a single protein. These results strongly suggest that *TALDO1* has two translational initiation sites, generating two distinct isoforms.

To confirm this theory, we constructed three single alanine substituted mutants, which contained the substitution at the first methionine codon (M1A), the second methionine codon (M11A), or the optimised first Kozak sequence (S2A) (Fig. 1D), each containing a C-terminal 3×HA tag, and the expression of each mutant was analysed by immunoblotting. As shown in Fig. 1E (left panel), the M1A mutation diminished the upper band and enhanced the expression of the lower band, while the M11A mutation abolished the lower band. The optimised first Kozak sequence (S2A) enhanced the expression of the upper band and nearly abolished the lower band. Together, these data indicate that the expression of the two different TALDO1 isoforms, a longer isoform, which we named TALDO1L, and a shorter one lacking the first 10 amino acids, named TALDO1S, is mediated by alternative translational initiation.

### The first 10 N-terminal amino acids of TALDO1 are essential for its nuclear localisation

**Figure 2:**
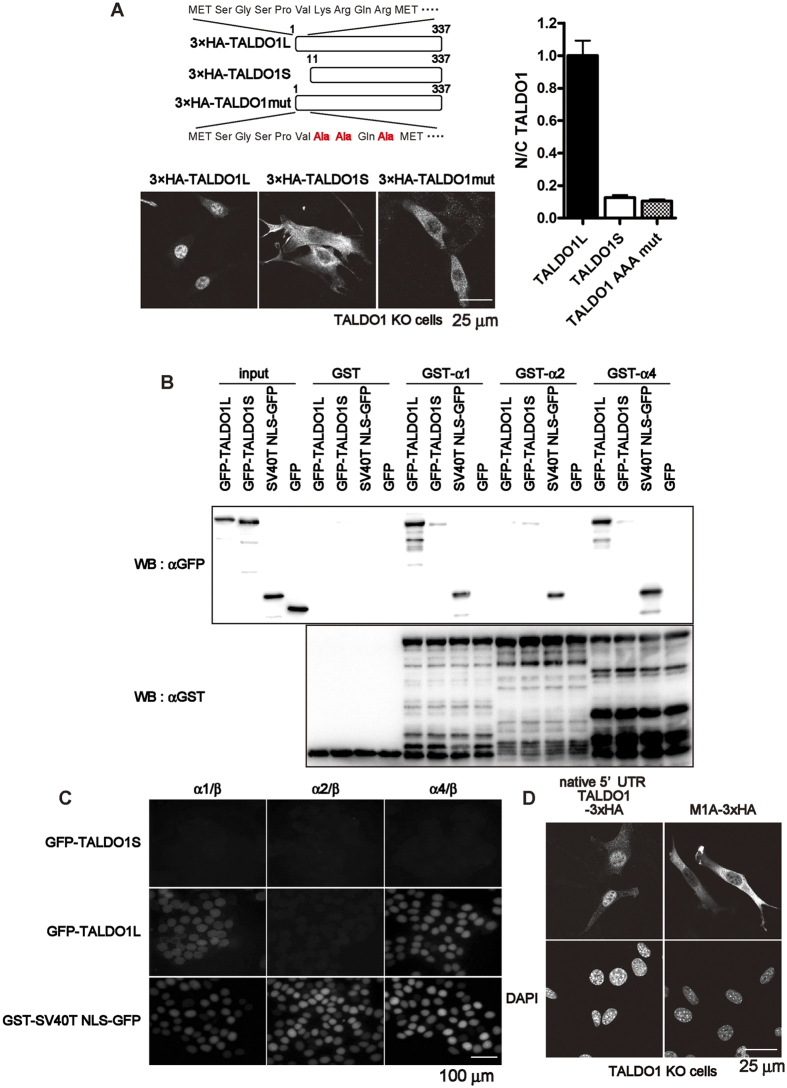
The 10 N-terminus amino acids are important for the nuclear localisation of TALDO1. (**A**) Immunofluorescence images of TALDO1 isoforms. TALDO1 KO cells were transiently transfected with vectors expressing N-terminally HA-tagged TALDO1 long isoform (TALDO1L, 1–337 amino acids), TALDO1 short isoform (TALDO1S, 11–337 amino acids), or TALDO1 K7A, R8A, and R10A mutant (TALDO1 AAA mut) and stained with anti-HA antibodies. Quantification of the nuclear-cytoplasmic (N/C) ratio of TALDO1 isoforms and mutants. The ratio was normalised to that of TALDO1L (mean ± S.E.M., *n* = 23 cells). (**B**) Importin α subtypes recognize TALDO1L but not TALDO1S. GST pull-down assays were performed using GST, GST-importin α1 (GST-α1), GST-importin α2 (GST-α2), or GST-importin α4 (GST-α4) with TALDO1-GFP isoforms or GFP (as the negative control). The bound proteins were analysed by western blotting with an anti-GFP antibody. Used GST-fusion proteins (1 μg) were analysed by Coomassie Brilliant Blue staining. Asterisks indicate the full-length GST-fusion proteins. (**C**) *In vitro* transport assay revealed that GFP-TALDO1L protein, but not GFP-TALDO1S protein, was transported into the nucleus in the presence of importin α, importin β, Ran-GDP, and an ATP regenerating system. GST-SV40T NLS-GFP was used as a positive control. (**D**) Immunofluorescence images of TALDO1 KO cells expressing either native 5′ UTR-TALDO1-3×HA or M1A-3×HA. The transfected cells were stained as described above.

Next, we determined the subcellular localisation of the two isoforms, 3×HA-TALDO1L and 3×HA-TALDO1S. To exclude the effect of the endogenous TALDO1, we transfected the isoform-specific expression vectors into TALDO1 KO cells. Surprisingly, these two isoforms showed different subcellular localisations; while 3×HA-TALDO1L was predominantly localised in the nucleus, the majority of 3×HA-TALDO1S was localised in the cytoplasm (Fig. 2A). The nuclear import of proteins is generally dependent on the presence of a specific signal sequence, the NLS, which is composed of basic amino acids. To determine whether the basic amino acids within the first 10 amino acids of TALDO1 are essential for its nuclear import, we constructed an AAA mutant, in which three basic amino acids within the 10 N-terminal amino acids were converted into alanines (K7A/R8A/R10A), and we transiently expressed it in TALDO1 KO cells. As expected, the AAA mutant was mainly distributed in the cytoplasm, similarly to 3×HA-TALDO1S. These results suggest that the first 10 amino acids of TALDO1 are essential for its nuclear localisation.

Our previous study showed that TALDO1L binds to mouse importin α1 (KPNA1)^3^. Therefore, we used a glutathione-S-transferase (GST) pull-down assay to test whether these TALDO1 isoforms are recognised by multiple importin α subtypes. As shown in Fig. 2B, we found that GST-importin α1 and GST-importin α4 (KPNA4) interacted with green fluorescent protein (GFP)-TALDO1L but not with GFP-TALDO1S. In contrast, GST-importin α2 (KPNA2) did not bind to either TALDO1 isoform. To examine whether these protein interactions reflect the nuclear localisation of TALDO1, we performed an *in vitro* transport assay. A GST-SV40T NLS-GFP fusion protein (GST-SV40T NLS-GFP) containing the NLS of the simian virus 40 large T-antigen was used as a positive control, and was efficiently transported into the nucleus in the presence of importin α and importin β. As shown in Fig. 2C, the nuclear import of GFP-TALDO1L, but not GFP-TALDO1S, was observed in the presence of TALDO1-interacting importin α isoforms together with importin β. Thus, these results indicate that importin α recognises TALDO1L through its 10 N-terminal amino acids, and actively transports it into the nucleus in an importin α/β-dependent manner.

We also analysed the subcellular localisation of native 5′ UTR TALDO1-3×HA and M1A-3×HA in TALDO1 KO cells (Fig. 2D). M1A-3×HA was predominantly localised in the cytoplasm, most likely corresponding to the distribution of TALDO1S (Fig. 2A). In contrast, the subcellular localisation of native 5′ UTR TALDO1-3×HA proteins was mostly nuclear. Collectively, these results indicate that the subcellular localisation of TALDO1 is determined by the translation initiation site used.

### TALDO1 forms both homo- and hetero-dimers

**Figure 3:**
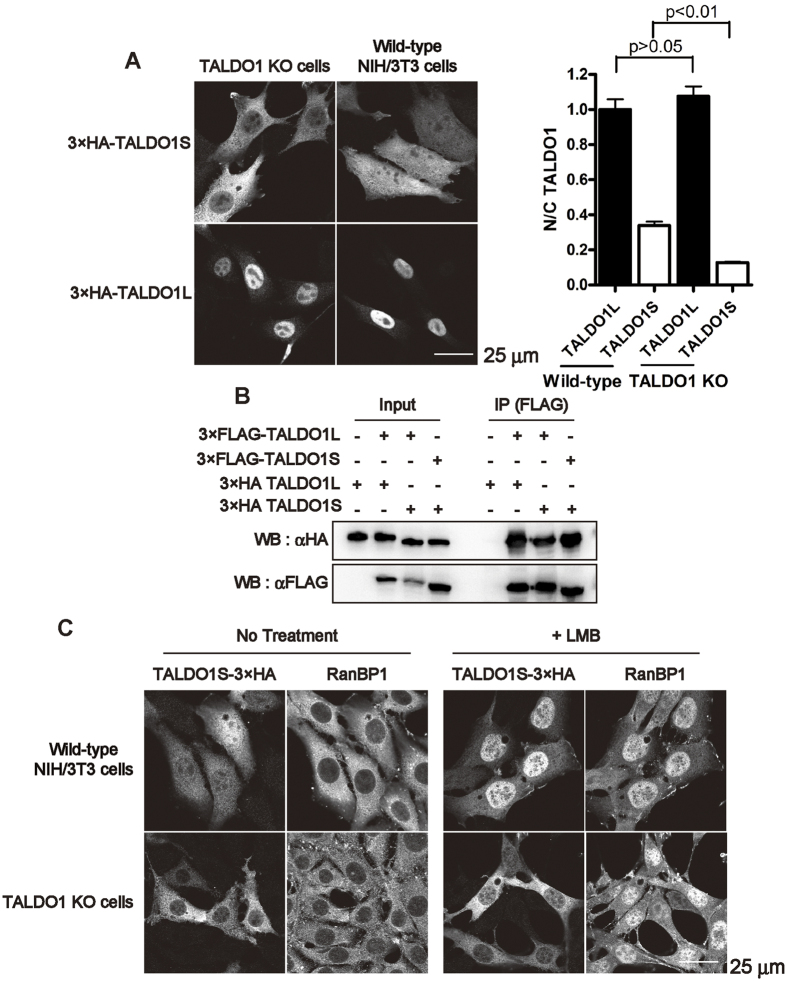
TALDO1 isoforms form hetero/homo dimers. (**A**) Immunofluorescence images of wild-type NIH/3T3 and TALDO1 KO cells transfected with either a 3×HA-TALDO1L or 3×HA-TALDO1S expressing plasmid. Quantification of the N/C ratio of TALDO1 isoforms. The ratio was normalised to that of TALDO1L in NIH/3T3 cells (mean ± S.E.M., *n* = 71 cells). *p*-values were calculated using one-way ANOVA with Tukey’s multiple comparison post-hoc test. (**B**) NIH/3T3 cell lysates were prepared from the cells transfected with 3×FLAG- or 3×HA-tagged TALDO1 isoforms or empty vectors, and used for immunoprecipitation with an anti-FLAG antibody. The bound proteins were analysed by western blotting with anti-HA and anti-FLAG antibodies. (**C**) The effects of leptomycin B (LMB) on the subcellular localisation of TALDO1S in wild-type NIH/3T3 and TALDO1 KO cells. The cells expressing TALDO1S-3×HA (M1A-3×HA) were treated for 6 h in the presence or absence of 10 nM LMB. LMB inhibited the nuclear export of endogenous RanBP1.

We noticed that overexpressed TALDO1S showed different subcellular distributions when expressed in either wild-type NIH/3T3 cells or TALDO1 KO cells (Fig. 3A); 3×HA-TALDO1S was predominantly localised in the cytoplasm in TALDO1 KO cells (as shown in Fig. 2A), whereas it was distributed throughout the nucleus and cytoplasm in wild-type NIH/3T3 cells. In contrast, the localisation of 3×HA-TALDO1L in wild-type NIH/3T3 cells was similar to that in TALDO1 KO cells. Since TALDO1 contains a conserved interface domain to form a dimer^20^, we speculated that endogenous TALDO1, majorly present as the TALDO1L isoform in NIH/3T3 cells, may form a heterodimer with exogenous TALDO1S, altering its subcellular distribution.

Therefore, to address the dimer-forming ability of TALDO1, two isoforms of TALDO1 harbouring different epitope tags (FLAG and HA) were transiently expressed in TALDO1 KO cells, and their interaction was analysed by co-immunoprecipitation using anti-FLAG antibodies. As shown in Fig. 3B, the immunoreactive band of 3×HA-TALDO1L was detected only in the presence, but not in the absence, of triple FLAG (3×FLAG)-tagged TALDO1L, demonstrating dimer formation between 3×HA-TALDO1L and 3×FLAG-TALDO1L (Fig. 3B, lane 6). Moreover, we found that the deletion of the first 10 amino acids did not interfere with the dimerisation potential of TALDO1 (Fig. 3B, lanes 7 and 8). These results suggest that the dimers containing the long isoform, namely TALDO1L homodimer and TALDO1L/TALDO1S heterodimer, may be actively imported into the nucleus.

Our previous study also demonstrated that full-length TALDO1 (TALDO1L) is exported into the cytoplasm by CRM1 (exportin 1)^3^. Using leptomycin B (LMB), a specific inhibitor of CRM1-dependent nuclear export^21^, we examined whether the short isoform of TALDO1 (TALDO1S-3×HA) is exported in a CRM1-dependent manner in wild-type NIH/3T3 cells or TALDO1 KO cells. The endogenous Ran binding protein RanBP1, which is exported in a CRM1-dependent manner, accumulated in nuclei after LMB treatment, as reported previously^22^. As shown in Fig. 3C, LMB treatment caused a drastic nuclear accumulation of TALDO1S-3×HA in wild-type NIH/3T3 cells (Fig. 3C), clearly demonstrating that its nuclear export was mediated by CRM1. In contrast, the cytoplasmic localisation of TALDO1S-3×HA in TALDO1 KO cells was unchanged after LMB treatment, suggesting that TALDO1 dimers do not migrate into the nucleus by passive diffusion. Since TALDO1S-3×HA potentially forms a heterodimer (TALDO1S-3×HA/endogenous TALDO1L) in wild-type NIH/3T3 cells, but not in TALDO1 KO cells, these results suggest that TALDO1 dimers containing the long isoform (TALDO1L/TALDO1L or TALDO1L/TALDO1S) can shuttle between the nucleus and the cytoplasm, while the homodimer of the short isoforms (TALDO1S/TALDO1S) is localised only in the cytoplasm.

### TALDO1 has different functions in the nucleus and the cytoplasm

**Figure 4:**
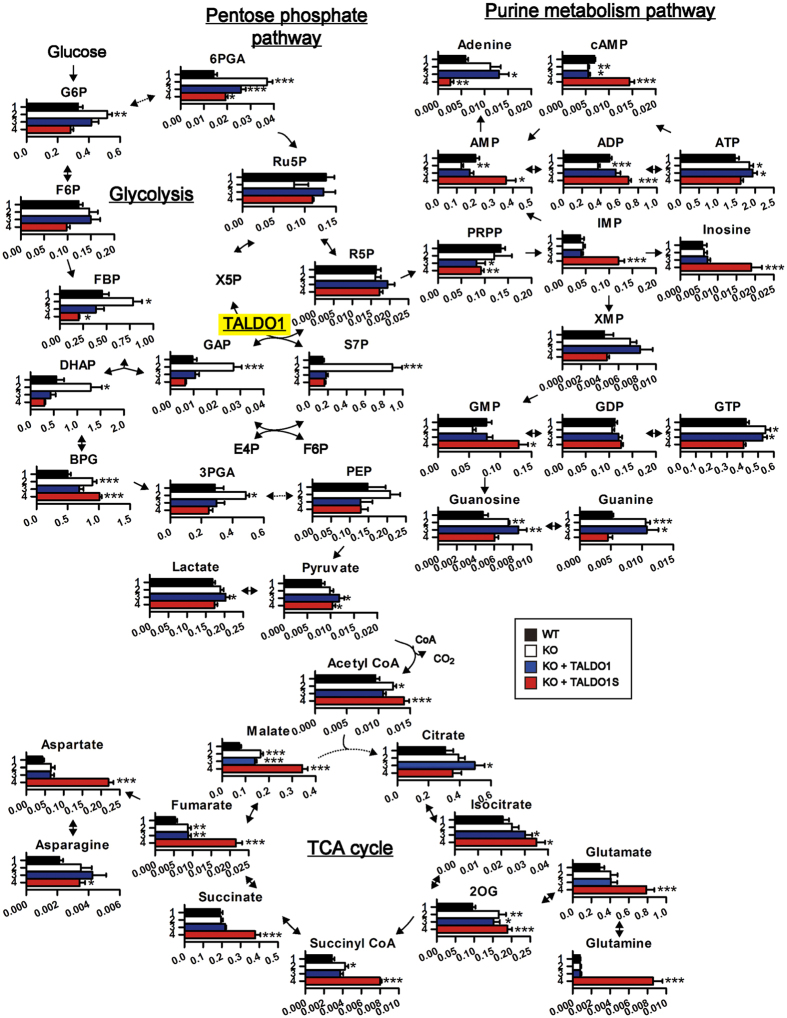
Metabolic profiling in wild-type and TALDO1 KO NIH/3T3 cells infected with empty, wild-type TALDO1, or TALDO1S expression retroviral vectors. (*p < 0.05, **p < 0.01, ***p < 0.005, two-tailed Student’s *t*-test).

Next, to assess how the differentially localised TALDO1 affects global metabolite levels, we prepared cell lysates from control wild-type NIH/3T3 cells (infected with empty retroviral vector) and TALDO1 KO cells infected with empty, wild-type TALDO1, or TALDO1S expression retroviral vectors (Supplementary Figure 2) and performed a metabolic analysis using high performance liquid chromatography with mass spectrometry (HPLC/MS). As shown in Fig. 4A and Supplementary Table 1, the lack of TALDO1 caused an approximately 6-fold upregulation of S7P, as previously reported^23^, verifying that our CRISPR/Cas9 procedure disrupted the function of TALDO1. The levels of GAP, a substrate of the forward reaction of TALDO1 as S7P, and the surrounding metabolic intermediates (FBP, DHAP, BPG, and 3PGA) were also significantly increased in the TALDO1 KO cells, indicating important roles of TALDO1 in the regulation of these metabolic intermediates of the pentose phosphate pathway. Notably, we found that the introduction of wild-type TALDO1 or TALDO1S induced the reduction of S7P, GAP, and the surrounding metabolic intermediates to a similar level as that observed in wild-type NIH/3T3 cells. Additionally, the level of 6PGA, which increased in TALDO1 KO cells, as described in a previous report^7^, decreased upon the expression of either wild-type TALDO1 or TALDO1S. These findings indicate that TALDO1 could function as a pentose phosphate pathway enzyme in both the nucleus and the cytoplasm.

However, unexpectedly, the analysis of additional metabolites revealed that the subcellular localisation of TALDO1 significantly influenced the levels of a broad range of metabolites in other pathways. In the purine metabolism pathway, IMP, GMP, AMP, inosine, and ADP significantly increased upon the expression of cytoplasm-localised TALDO1S, but not with wild-type TALDO1. Notably, the expression of cytoplasmic TALDO1S also caused an increase in the levels of a broad range of metabolites in the TCA cycle, including acetyl-CoA, succinyl-CoA, succinate, fumarate, malate, glutamate, glutamine, and aspartate. It should be mentioned that the reintroduction of *TALDO1* into TALDO1 KO cells only partially rescued the levels of some metabolites (e.g. malate, fumarate, and 2-oxoglutarate), which could be due to the relatively short time-course of the experiment (24 h after infection) or, alternatively, the excess amount of exogenous TALDO1 isoforms as compared with the endogenous one (data not shown). These results strongly suggest that TALDO1 not only functions as a pentose phosphate pathway enzyme, but also affects other metabolite pathways by altering its subcellular localisation between the nucleus and the cytoplasm.

## Discussion

In this study, we have demonstrated that two isoforms of TALDO1 proteins, which are generated by alternative translational initiation, differ in their nucleocytoplasmic localisations due to the availability of a functional NLS, and have significantly different effects on a broad range of metabolic pathways including not only the pentose phosphate pathway, but also glycolysis, the TCA cycle, and purine metabolism.

Alternative translational initiation has been reported to affect the nucleocytoplasmic distribution of proteins (e.g. progesterone receptor^24^, FGF2^25^, and the phosphoprotein of rabies virus^26^) or their biological roles (e.g. c-MYC^27^, BAG-1^28^, SCL^29^, and Orai1^17^). However, TALDO1 is, to our knowledge, the first example of a protein in which alternative translational initiation affects the levels of such a broad range of metabolites. The two in-frame translation initiation sites of the *Taldo1* gene, which are composed of the first AUG (containing a sub-optimal Kozak sequence) and the second AUG (containing a perfect Kozak sequence), are conserved among human, mouse, chicken, and zebrafish, but not in fly, nematode, or yeast (Fig. 1C). This implies that the regulation of the alternative translational initiation of *Taldo1* might be important for physiological function in vertebrates. Notably, the two isoforms of the TALDO1 protein were detected in several human cell lines, which showed different ratios of the long and short isoforms (Fig. 1A). Thus, the alternative translational initiation of this gene may be regulated in a cell type-specific manner.

Our metabolic analysis demonstrated that, surprisingly, both nuclear TALDO1L (long isoform) and cytoplasmic TALDO1S (short isoform) function as pentose phosphate pathway enzymes in a similar fashion. Notably, cytoplasmic TALDO1 affected the levels of metabolites involved in glycolysis, purine metabolism, and the TCA cycle to a greater extent than nuclear TALDO1 did (Fig. 4). Although the mechanism by which the cytoplasmic TALDO1 more efficiently affects metabolite levels is unknown, we speculate that cytoplasmic TALDO1 might directly bind to metabolic enzyme(s) or regulator(s) of other metabolic pathways to affect their function. In the future, it will be important to understand the molecular mechanism by which the altered subcellular distribution of the metabolic enzymes affects such a broad range of cellular metabolic pathways.

Additionally, we examined the subcellular localisation of other GFP-fused enzymes of the pentose phosphate pathway (Supplementary Figure 3). As expected, most of the pentose phosphate pathway enzymes were either localised in the cytoplasm (G6PDH, 6PGDH, and RPI) or distributed throughout the cell (6PGL and RPE), except for transketolase (TKT), which was predominantly localised in the nucleus. Thus, TKT could have differential functions depending on its subcellular localisation, like TALDO1.

Consistently, it has been shown that subsets of metabolic enzymes play differential roles depending on their subcellular localisation. For example, while the isoform of fumarase functions as a metabolic enzyme within the TCA cycle in mitochondria^30^,^31^, it also acts as a DNA damage response protein when recruited into the nucleus, following DNA damage^32^. Another example is the hexokinase (HK) family of enzymes, which catalyse the conversion of glucose to glucose 6-phosphate, the first step of glycolysis. Two HK isoforms, HKI and HKII, show different subcellular localisations^33^. It has been shown that the functional differences between these HK isoforms are mainly attributed to their subcellular localisation; while HKs associated with mitochondria promote glycolysis, those situated in the cytoplasm promote glycogen synthesis^34^. Additionally, aconitase participates in two different metabolic pathways depending on its subcellular localisation^35^. Moreover, it has been reported that pyruvate kinase M2 (PKM2), an isoform of pyruvate kinase involved in aerobic glycolysis^36^,^37^, is transported into the nucleus when phosphorylated^38^, and functions as a transcriptional coactivator that promotes glycolysis in cancer cells^39^,^40^,^41^. These results, together with our observations, indicate the functional importance of the subcellular localisation of metabolic enzymes. In summary, our study has demonstrated that the function of TALDO1, which has critical roles in a broad range of metabolic pathways, is finely regulated by several mechanisms including alternative translational initiation, active nucleocytoplasmic transport, and dimer formation.

## Methods

### Plasmid construction

Human and mouse native 5′ UTR TALDO1 and TALDO1 short isoform were generated by polymerase chain reaction (PCR) amplification from total RNA from mouse brain, embryonic stem cells, or HEK293 cells using appropriate primers (Supplementary Table 2), and subsequently subcloned into pcDNA3.1Zeo(+) (Thermo Fisher Scientific, Waltham, MA), pEGFP-N1 (Takara Bio Inc., Shiga, Japan), pGEX-6p-2 (GE Healthcare Japan, Tokyo, Japan), p3×HA-CMV DEST^42^, p3×FLAG-CMV DEST^43^, and pcDNA3.1Zeo(+) 3×HA C1 DEST vectors, which were each created via the insertion of three tandem repeats of the HA oligonucleotide: 5′-TACCCATACGACGTCCCAGACTACGCTTACCCATACGACGTCCCAGACTACGCTTACCCATACGACGTCCCAGACTACGCT-3′ into the PstI and XhoI sites, and the Reading Frame Cassette of the Gateway^®^ Conversion System (Thermo Fisher Scientific) into the EcoRV site of the pcDNA3.1Zeo(+) vector. TALDO1 mutants were constructed by site-directed mutagenesis and by inverse PCR mutagenesis with the KOD-plus, Ligation High ver. 2, and T4 polynucleotide kinase enzymes (TOYOBO, Osaka, Japan) using primers (Supplementary Table 2).

For the cloning of TALDO1 KO cell lines using the CRISPR-Cas9 system, target sequences were designed according to the CRISPR Design Tool (http://crispr.mit.edu/, Supplementary Table 2) and the corresponding annealed oligonucleotides were subcloned into the BbsI (BpiI) sites of pSpCas9 (BB)-2A-Puro (Addgene, Cambridge, MA). Retroviral expression vectors for TALDO1 and TALDO1 short isoform were constructed by subcloning the fragments of TALDO1 and TALDO1 short isoform, respectively, into the BamHI and XhoI sites of pMXs (Addgene).

### Cell culture

HepG2, Hep3B, HLE, HLF, HuCCT1, and HuH28 cells were obtained from the Japanese Collection of Research Bioresources (Osaka, Japan). Plat-E cells were obtained from Cell Biolabs Inc. (San Diego, CA). HeLa, NIH/3T3, TALDO1 KO, HepG2, Hep3B, HCE, and HCF cells were cultured in Dulbecco’s modified Eagle’s Medium (DMEM; Sigma-Aldrich, St. Louis, MO) supplemented with 10% foetal bovine serum (FBS). Plat-E cells were cultured in DMEM containing 10% FBS, puromycin (1 μg/mL), and blasticidin S (10 μg/mL). HuCCT1 and HuH28 were cultured in RPMI-1640 (Sigma-Aldrich) supplemented with 10% FBS. The cells were maintained in a 37 °C incubator with 10% CO_2_-humidified air.

### Transient transfection

Transfection was performed using Lipofectamine^®^ 2000 (Thermo Fisher Scientific) according to the manufacturer’s instructions.

### Immunofluorescence

NIH/3T3 cells were plated at 2.5 × 10^5^ cells per well in 6-well plates with coverslips and cultured for 24 h. The cells were fixed with 3.7% formaldehyde for 10 min at room temperature. After washing with PBS, the cells were treated with ice-cold methanol for 10 min at −30 °C. After blocking with Blocking One Histo (Nacalai Tesque, Kyoto, Japan) and washing with PBS containing 0.05% Tween^®^ 20, the cells were incubated with primary antibodies overnight at 4 °C. After washing with PBS, Alexa Fluor^®^ 488- or 594-conjugated secondary antibodies (Thermo Fisher Scientific) were used. Nuclei were stained using 0.1 μg/mL 4′,6-diamidino-2-phenylindole (DAPI) (Dojindo, Kumamoto, Japan). The fluorescence was observed by a Zeiss Axioplan 2 fluorescence microscope (Carl Zeiss, Oberkochen, Germany) and a Leica TCS SP8 microscope (Leica Microsystems, Wetzlar, Germany).

### Expression and purification of recombinant proteins

GST fusion proteins were expressed in *Escherichia coli* strain BL21 and purified. Briefly, the cells were grown to an OD600 of 0.6 at 37 °C, and the expression of GST-fusion proteins was induced with 0.1 mM IPTG at 18 °C for 16 h. After inducing the expression of GST fusion proteins, the cells were harvested and resuspended in bacterial lysis buffer (50 mM Tris-HCl [pH 8.3], 500 mM NaCl, 1 mM EDTA, 2 mM DTT, and 0.2 mM PMSF). For purification of the GST fusion proteins, the crude lysates were incubated with Glutathione Sepharose™ 4B (GE Healthcare) at 4 °C for 2 h. The protein-bound beads were incubated with elution buffer (100 mM Tris-HCl [pH 8.3], 100 mM NaCl, 1 mM EDTA, 2 mM DTT, 1 μg/mL aprotinin, 1 μg/mL leupeptin, and 1 μg/mL pepstatin). The eluted proteins were desalted using a PD-10 column (GE Healthcare) that was equilibrated in transport buffer (TB; 20 mM HEPES [pH 7.3], 110 mM potassium acetate, 2 mM magnesium acetate, 5 mM sodium acetate, 0.5 mM EGTA, 2 mM DTT, 1 μg/mL aprotinin, 1 μg/mL leupeptin, and 1 μg/mL pepstatin). For the *in vitro* transport assay, GST fusion proteins were incubated with a PreScission™ Protease (GE Healthcare) and GST tag-cleaved fusion proteins were purified.

### GST pull-down assay

Test proteins (50 pmol) were incubated with 50 pmol each of GST, GST-mouse importin α1, GST-mouse importin α2, or GST-mouse importin α4 immobilized on Glutathione Sepharose™ 4B beads (GE Healthcare) in binding buffer (20 mM HEPES [pH 7.3], 150 mM NaCl, 0.5% NP-40, 1 mM DTT, 1 μg/mL aprotinin, 1 μg/mL leupeptin, and 1 μg/mL pepstatin) containing 1% bovine serum albumin at 4 °C for 2 h. After the beads were washed with the binding buffer, they were resuspended in sodium dodecyl sulphate (SDS) sample buffer. The purified protein samples were subjected to SDS-polyacrylamide gel electrophoresis and then western blotting was performed using anti-GFP or anti-GST antibodies.

### Antibodies

Anti-FLAG M2 (1:1000 dilution; F1804, Sigma-Aldrich), anti-HA (1:1000 dilution; 11-867-423-001, Roche, Mannheim, Germany), anti-GFP (1:2000 dilution; A11055, Thermo Fisher Scientific), anti-GST (1:2000 dilution; sc-138, Santa Cruz Biotechnology, Inc., Dallas, TX), anti-TALDO1 (1:1000 dilution; sc-51440, Santa Cruz Biotechnology), and anti-actin (1:1000 dilution; sc-1615, Santa Cruz Biotechnology) antibodies were used for immunoblotting. For immunofluorescence, antibodies against TALDO1 (1:100 dilution; sc-51440, Santa Cruz Biotechnology or 1:200 dilution; HPA040373, Sigma-Aldrich), anti-HA antibody (1:300 dilution; 11-867-423-001, Roche Diagnostics, Mannheim, Germany), and RanBP1 (1:100 dilution; sc-28576, Santa Cruz Biotechnology) were used.

### *In vitro* transport assay

*In vitro* transport assay was performed as described previously^42^. Briefly, HeLa cells were cultured in a 100-mm dish with an 8-well slide (MP Biomedicals, Inc., Santa Ana, CA). The next day, after washing with ice-cold TB, the cells were permeabilised with 40 μg/mL digitonin (Nacalai Tesque) in TB on ice for 5 min. Then, after a further wash with ice-cold TB, the cells were immersed in ice-cold TB for 10 min. The slide was then immersed in TB containing TALDO1S-GFP, TALDO1L-GFP, or GST-SV40NLS-GFP, and incubated at 30 °C for 30 min. Finally, the cells were rinsed with ice-cold TB and then fixed with 3.7% formaldehyde in TB for 15 min. GFP-tagged proteins were observed with a Zeiss Axioplan 2 fluorescence microscope (Carl Zeiss).

### Generation of TALDO1 KO cells using the CRISPR-CAS9 system

TALDO1 target sequence (64-ACCACCGTGGTGGCCGACAC-83), guide sequence, sgRNA scaffold, and Cas9 nuclease derived from *Streptococcus pyogenes* were expressed in NIH/3T3 cells by transfecting them with the constructed pSpCas9(BB)-2A-Puro using Lipofectamine^®^ 2000. Puromycin selection was carried out at 24 h post-transfection. NIH/3T3 cells were cultured in DMEM containing 3 μg/mL puromycin for 3 days, and re-cultured in fresh DMEM until the formation of colonies was observed. Genomic changes were identified using genomic PCR against off-target genes (*Wbscr16*, *Ikzf2*, *Thsd1*, and *Ttc28*; the primers are listed in Supplementary Table 2), and using DNA sequence against *Taldo1*, which was subcloned into a mammalian expression vector, to detect two genomic changes in a clonal cell line. We confirmed the KO of TALDO1 using western blotting and immunofluorescence.

### Retroviral infection

Plat-E cells were plated at a density of 3.0 × 10^5^ cells in a 100-mm dish and cultured for 24 h. Using Lipofectamine^®^ 2000, the cells were transfected with pMXs, pMXs-TALDO1, or pMXs-TALDO1 short isoform. The viral supernatants were harvested at 48 h post-transfection and filtered through a 0.45-μm membrane, followed by an incubation with 8 mg/mL hexadimethrine bromide. The mixed media were transferred to the NIH/3T3 or TALDO1 KO cells, which were plated at a density of 1 × 10^6^ cells in a 100-mm dish and cultured for 24 h.

### Sample preparation for HPLC/MS

The infected NIH/3T3 or TALDO1 KO cells were washed twice with PBS and subsequently harvested using a cell lifter (Corning Inc., Corning, NY), before transferring them into a 2 mL safe-lock tube (Eppendorf, Hamburg, Germany). We immediately centrifuged the tubes at 3000 × *g* for 3 min at 4 °C and discarded the supernatant. The cell pellets were frozen with liquid nitrogen and stored at −80 °C until lyophilisation.

The lyophilised cell pellets were weighed for normalisation and homogenised using a MM 301 mixer mill (Retsch GmbH, Haan, Germany) with zirconium balls at 20 Hz for 1 min. The cell homogenate was resuspended in 1 mL ice-cold extraction buffer (methanol:water:chloroform, 5:2:2) and shaken at 1300 rpm for 30 min at 4 °C. Four hundred microlitres of H_2_O were then added to the cell extract and the mixture was centrifuged at 13000 rpm for 5 min at 4 °C. We filtered the supernatant with a Millex^®^-LG 0.2-μm PTFE filter (Millipore, Billerica, MA), followed by concentration to remove the organic solvent. The sample was lyophilised and redissolved in 50 μL distilled water.

### HPLC/MS analysis

HPLC/MS was performed on a LCMS-8030+ (Shimadzu, Kyoto, Japan) with an L-column2 ODS (2.1 × 150 mm, 3 μm; Chemicals Evaluation and Research Institute, Oita, Japan). In chromatographic analysis, tributylamine was used as an ion pair reagent and the sample was injected at a 0.3 mL/min flow rate, with a 40 °C column temperature and a total injection volume of 3 μL. The chromatographic gradient separation was achieved by mixing mobile phase A (15 mM tributylamine and 10 mM acetic acid) and mobile phase B (methanol) as follows; 0% B in A, 0–0.5 min; 25% B in A, 0.5–7.5 min; 100% B in A, 7.5–12 min; 100% B in A, 12–13 min; 0% B in A, 13–14 min; and 0% B in A, 14–16 min. Electrospray ionisation was carried out in negative ion mode and mass spectrometric analysis was performed under the following conditions: probe position, +1.5 mm; desolvation lines temperature, 250 °C; nebuliser gas flow, 2 L/min; heat block temperature, 400 °C. Other conditions were determined by the instrument’s auto-tuning program.

Using LabSolutions LC/MS (Shimadzu), we detected mass spectral peaks in multiple-reaction-monitoring mode and identified the metabolites based on the retention times of standard samples. The area values of each metabolite were divided by the area value of piperazine-1,4-*bis*(2-ethanesulphonic acid), which was used as an internal control for baseline correction, and then divided by the weight of the lyophilised cell pellet for normalisation. Multivariate analysis was performed using the Umetrics SIMCA-P+ 12 software program (MKS Data Analysis Solutions, Umeå, Sweden).

## Additional Information

**How to cite this article**: Moriyama, T. *et al*. Two isoforms of TALDO1 generated by alternative translational initiation show differential nucleocytoplasmic distribution to regulate the global metabolic network. *Sci. Rep.* **6**, 34648; doi: 10.1038/srep34648 (2016).

## Supplementary Material

Supplementary Information
