# Supplementary material for: Two isoforms of TALDO1 generated by alternative translational initiation show differential nucleocytoplasmic distribution to regulate the global metabolic network
Source: Sci Rep. 2016 Oct 5;6:34648. doi: 10.1038/srep34648 (PMC5050407; doi:10.1038/srep34648)
Supplement: Supplementary Information [file srep34648-s1.doc]

**Supporting Information**

**Two isoforms of TALDO1 generated by alternative translational initiation show differential nucleocytoplasmic distribution to regulate the global metabolic network**

**Supplementary Figure 1-3**

**Supplementary Table 1 and 2**

**
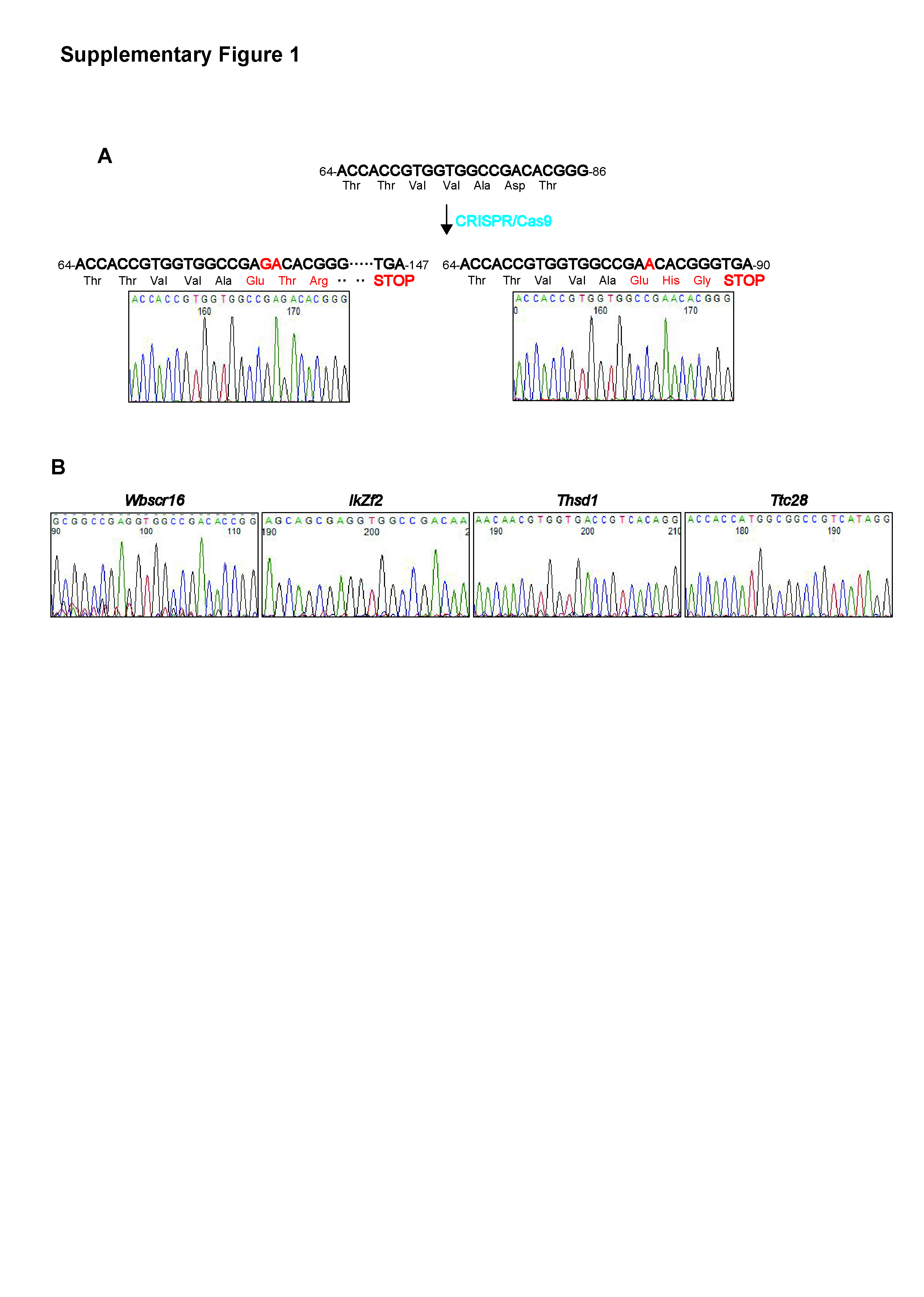
**

S**upplementary Figure 1**. TALDO1 KO cells were generated using the CRISPR-Cas9 system. (A) Genomic sequences of *Taldo1* in the cloned cell lines. DNA sequence analysis identified insertions near the proto-spacer adjacent motif (GGG). (B) Genome sequences of off-target candidates. Genomic PCR analyses of *Wbscr16* (Williams–Beuren syndrome chromosome region 16 homolog), *Ikzf2* (IKAROS family zinc family 2), *Thsd1* (thrombospondin, type1), and *Ttc28* (tetratricopeptide repeat domain 28) confirmed that genome editing using the CRISPR-Cas9 system did not change their genomic sequences.


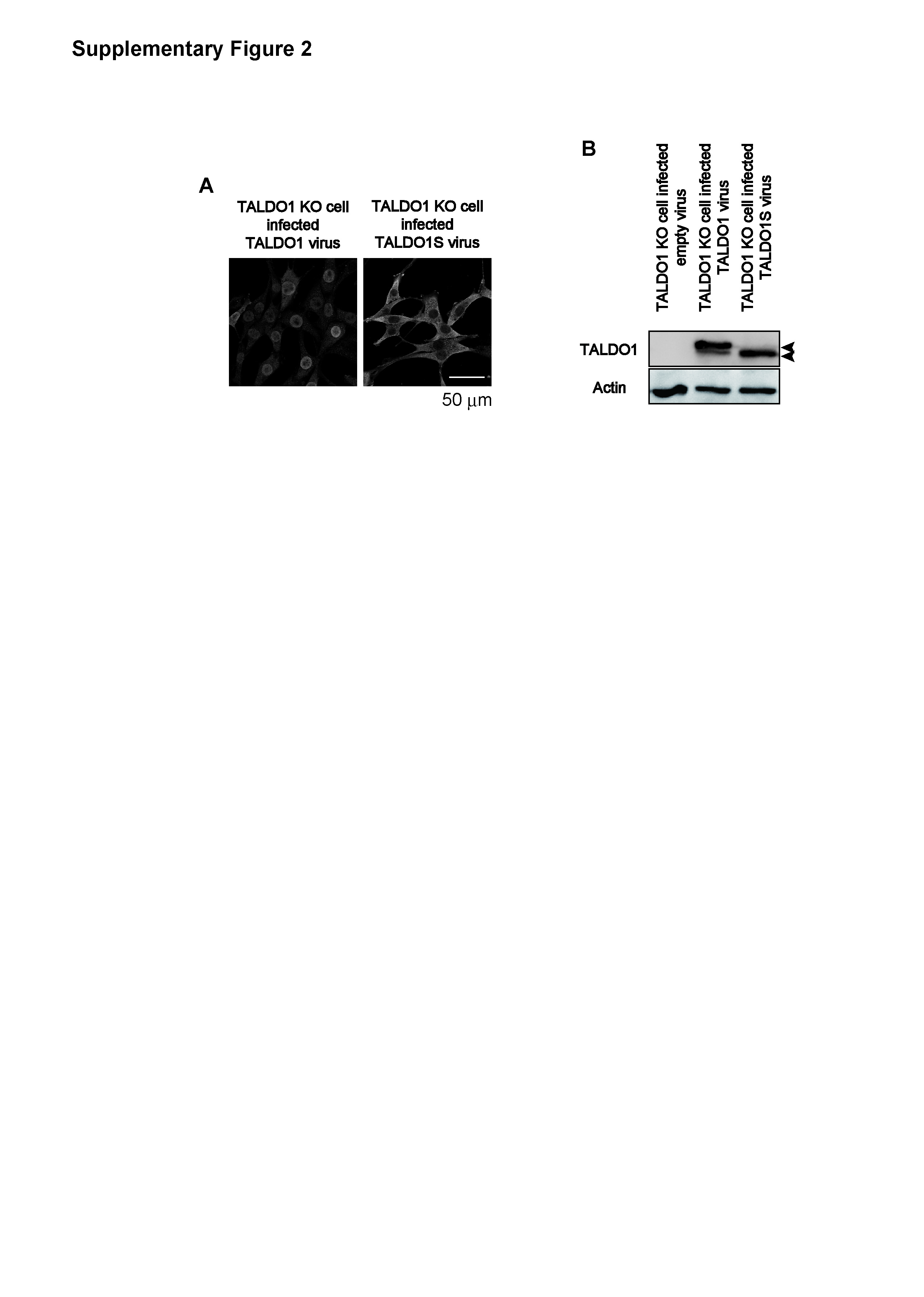


S**upplementary Figure 2**. Western blot and immunofluorescence analysis of TALDO1 isoforms in cell lines infected with TALDO1 or TALDO1S expression retroviral vector.

(A) TALDO1 KO cells infected with TALDO1 or TALDO1S expression retroviral vector were stained with anti-TALDO1 antibodies and (B) analysed by western blotting with anti-TALDO1 and anti-actin antibodies.


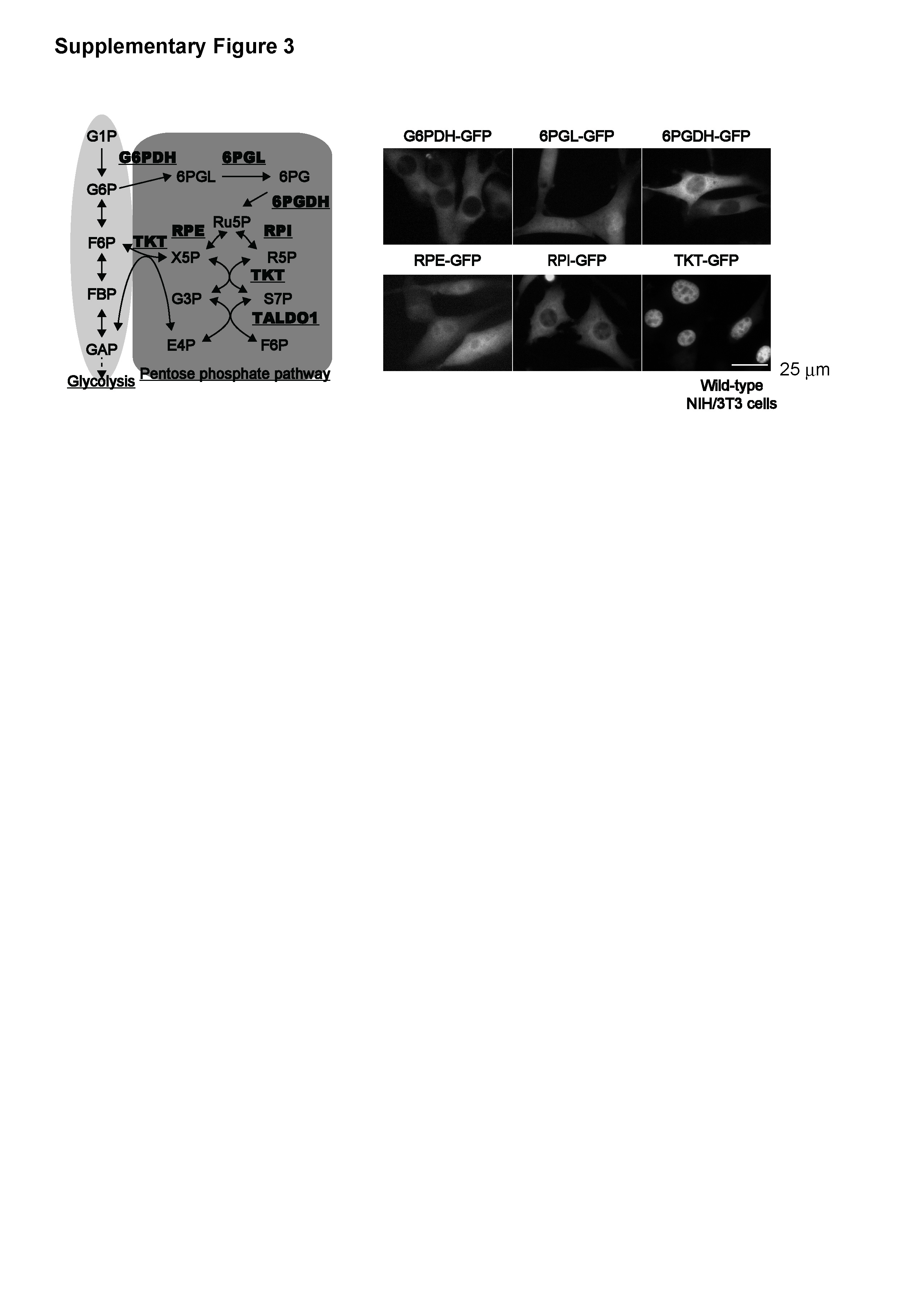


S**upplementary Figure 3**. Subcellular localisation of metabolic enzymes involved in the pentose phosphate pathway. NIH/3T3 cells were transiently transfected with the indicated GFP-fusion protein expressing vectors, and observed. G6PDH, glucose-6-phosphate dehydrogenase; 6PGL, 6-phosphogluconolactonase; 6PGDH, 6-phosphogluconate dehydrogenase; RPE, ribulose-5-phosphate 3-epimerase; RPI, ribulose-5-phosphate isomerase; TKT, transketolase.

S**upplementary Table 1**.

Measurement of metabolites involved in central carbon metabolism in wild-type NIH/3T3 or TALDO1 KO cells infected with empty, TALDO1, or TALDO1S expression retroviral vectors.

S**upplementary Table 2**.

Primers used in this study.
